# Hepatitis C Infection and Periodontal Disease: Is there a Common Immunological Link?

**DOI:** 10.1155/2018/8720101

**Published:** 2018-03-13

**Authors:** Dorin Nicolae Gheorghe, Liliana Foia, Vasilica Toma, Amelia Surdu, Elena Herascu, Dora Maria Popescu, Petra Surlin, Cristin Constantin Vere, Ion Rogoveanu

**Affiliations:** ^1^Department of Periodontology, Faculty of Dental Medicine, University of Medicine and Pharmacy of Craiova, 2 Petru Rares street, 200349 Craiova, Romania; ^2^Department of Biochemistry, University of Medicine and Pharmacy “Grigore T. Popa”, 16 Universitatii street, 700115 Iasi, Romania; ^3^Department of Oral Surgery, University of Medicine and Pharmacy “Grigore T. Popa”, 16 Universitatii street, 700115 Iasi, Romania; ^4^Department of Implantology, University of Medicine and Pharmacy “Grigore T. Popa”, 16 Universitatii street, 700115 Iasi, Romania; ^5^Department of Gastroenterology, Faculty of Medicine, University of Medicine and Pharmacy of Craiova, 2 Petru Rares street, 200349 Craiova, Romania

## Abstract

Hepatitis C virus (HCV) infections could have an important impact on the oral health status of patients, favoring conditions such as periodontal disease and oral cancer. The review of the existing scientific literature written in English was performed, searching for oral and periodontal manifestations of HCV infection and its impact on the oral fluids. HCV infection can determine direct extrahepatic manifestations at the oral and periodontal level including oral lichen planus, Sjögren-like sialadenitis, and oral cancer. The changes caused by the infection in the subjects' immune system, diet, and lifestyle can facilitate the development of oral conditions such as periodontal disease. Important changes also occur in the composition of the infected patients' saliva and gingival fluid. HCV-infected patients need to be carefully monitored in terms of oral health since the infection with the virus can result in oral complications. The cellular and molecular particularities of the gingival fluid of HCV-infected patients can answer some questions regarding its impact upon periodontium impairment and whether this refers to a possible bidirectional relationship, with hepatic biomarker adjustments being induced by the periodontal patients' inflammatory status.

## 1. Introduction

Despite important efforts being made in the past years to raise awareness over transmission and treatment possibilities, hepatitis C virus (HCV) remains an important global health issue. The untreated viral infection leads to chronic inflammation of the liver. Chronic hepatitis has complications such as hepatic cirrhosis and hepatocellular carcinoma that are fatal for the patient [1, 2]. Moreover, the pathological events that HCV infection triggers expand beyond the liver, a number of extrahepatic manifestations including cryoglobulinemia, malignant lymphoma, Sjögren syndrome, or oral lichen planus having been already discussed in the literature [3–7].

Current data available from the WHO (World Health Organization) shows that, in 2015, more than 70 million people were HCV infected, resulting in a global prevalence of about 1%. The HCV infection affects all regions of the world, with important differences between countries. According to the WHO, the highest prevalence of HCV infection was reported for Eastern Mediterranean and European regions. An estimated 1.75 million new HCV infections occurred worldwide in 2015 [8]. The infection is difficult to diagnose in its early stages since it does not exhibit obvious clinical signs and can only be pinpointed through serological test. This aspect can be improved with a more careful analysis of extrahepatic manifestations that almost 75% of the infected patients express [9]. It is believed that the virus can be hosted inside extrahepatic tissues, making it more prone to transmission and more difficult to treat [10]. Hence, the development of viral infection complications such as hepatocellular carcinoma can have a critical impact on the patient's immune system [11], making it even more difficult to be tackled by the defensive physiological mechanisms.

The impact of HCV infection on oral health has received different opinions over time, varying from clinical evidence to meta-analysis and further wider approach on the subject [12]. Oral health impairment for the infected patients could be the result of liver malfunction, a damaged immune system, or the low drive of the infected patients to seek dental care [13, 14]. Several studies address the significance of HCV infection on the oral cavity, highlighting both the dental pathological changes and other extrahepatic manifestations (EHMs) with oral implications [15], while others reveal the impact type A, B, and C hepatitis has on oral fluids, spotting the possible carriage of the hepatitis viruses in the whole saliva and gingival fluid [16]. A more recent review of Han et al. points out the synergy between periodontal and liver conditions, other than viral hepatitis, outlining the possible joint impact that periodontal and hepatic inflammation could provide [17]. The present review article focuses mainly on the bidirectional relationship of periodontal inflammation and HCV infection in terms of clinical manifestations, the molecular expression of biomarkers within oral fluids and its significance for medical practitioners, both periodontists and hepatologists, with a subsequent interest upon whether the presence of periodontal inflammation could enhance the risk of hepatitis C virus transmission through the oral fluids, especially the gingival crevicular fluid (GCF).

## 2. HCV Infection: Impact on Oral Health and Periodontal Status

### 2.1. HCV Infection and Periodontal Status

Coates et al. studied the oral health issues of HCV-infected persons in a study upon 87 patients ageing between 35 and 44 yo [13]. The dental pathological changes, expressed with the help of the DMFT index (Decayed, Missing, Filled Teeth index), were three times more important for HCV-infected patients than for the control group for teeth with carious lesions. HCV-positive patients also expressed a larger number of missing teeth but had less dental fillings than the control [13].

The periodontal status of the patients was evaluated using the CPITN index (Community Periodontal Index of Treatment Needs), recording increased gingival bleeding for infected patients and deeper periodontal pockets, for the age groups ranging between 25–34 and 35–44 years. Even though the findings were not statistically significant for the number of subjects, a trend for impaired periodontal status has been pointed out in infected subjects. The authors state that diet characteristics and other social particularities such as lack of proper dental and periodontal treatment could be the cause of this trend [13].

A possible pathological mechanism that might shed some light upon the influence of chronic hepatitis C on periodontal status could be linked to insulin resistance (IR) and the development of a chronic inflammatory process in the liver.

Insulin resistance is a pathological condition in which cells do not react normally to insulin, being closely related to diabetes and obesity. Periodontal disease has been associated with the metabolic syndrome (including insulin resistance) [18], and a connection between HCV infection and insulin resistance has been validated as well [19]. It was suggested that, given the association between hepatic chronic inflammation and insulin resistance [19], the periodontal inflammatory response could be possibly modified in HCV patients with liver fibrosity and obesity [20]. Serfaty et al. suggested that multiple mechanisms seem to be involved in IR exacerbation in chronic HCV, the metabolic syndrome and chronic inflammatory processes being amongst them. These pathological pathways boost proinflammatory cytokines production (mainly TNF-alpha, adiponectin, and IL-6) [19]. These cytokines are involved either in periodontal disease triggering and progression or in the connection between periodontal disease and IR [21].

The liver controls many aspects of the immune system's physiology from defensive cell production to the control of the body's nonspecific immune activity leading to a chronic inflammatory state. As a result, the impaired hepatic function caused by chronic inflammation shifts the entire defensive abilities of the organism. Such changes can interest neutrophil cell characteristics (lower adherence, mobility, and phagocytic abilities) and the activity of the complement system which enhances the antibody-cell defensive reaction [22]. The collaboration between neutrophil cells and the complement system is extremely important for the normal activity of the defensive mechanisms against periodontal pathogenic bacteria [23].

Periodontal disease occurs when the ecological balance of the oral cavity is disrupted and periodontal bacterial pathogens start triggering an inflammatory reaction. Subsequently, the inflammation becomes chronic and causes the periodontal tissues' dissolution. The pathological mechanism of the disease can be influenced by impairment of the patient's immune system. This not only causes a shift in oral bacterial species but it also creates an inadequate inflammatory reaction [24]. This can be the case for patients suffering from leukemia or cancer or for those undergoing chemotherapy, as well as for patients with damaged liver function caused by liver cirrhosis [25].

The combination of internal factors such as insulin resistance, immunological dysfunction, ongoing chronic inflammatory status, and external ones including the lack of proper dental care could make the HCV-infected patients face the risk of periodontal disease development. In this aspect, not only do HCV-infected patients hesitate to address their dental issues but their treatment plan and options can also be limited by the unwillingness of some dentists to treat them due to the risk of infection spreading [26].

### 2.2. EHMs with Oral Implications and Periodontal Disease

While the dental and periodontal issues seem to be an indirect consequence of the viral infection, other oral health conditions such as oral lichen planus, Sjögren-like sialadenitis, and oral-squamous-cell carcinoma are directly caused by the immune deficiency caused by the virus, thus being considered extrahepatic manifestations (EHM) of the infection [15, 27, 28]. However, the biological mechanism through which the HCV infection could trigger oral extrahepatic manifestations has not yet been fully elucidated.

Lichen planus is a skin condition which manifests itself with inflammatory lesions. Lichen planus can also involve the oral mucosa, especially the interior lining of the cheeks, in this case being named oral lichen planus (OLP). In contrast to the skin location of the condition, where it often disappears spontaneously, the special local conditions of the oral cavity prevent this from happening and can even elicit the lesion to develop a malignant potential [29]. Meta-analyses reviews on the link between OLP and HCV infection have proven the existence of a strong connection between the two [30]. These findings have important clinical significance as the bidirectional connection between the two conditions is of interest for patients diagnosed with one or another, in order to be referred to both specialists. A study by Azizi and Rezaee showed that if the OLP lesion is positioned on the gingiva, it produces a form of desquamative gingivitis, with a negative impact on the periodontal status [31]. Clinically, this translates into a worsening of the studied indexes for the OLP patients versus the control, such as plaque index, gingival index, probing depth, bleeding on probing, and attachment loss [31]. The negative impact of OLP lesions on the periodontal status is also confirmed by a study of Lopez-Jornet and Camacho-Alonso that revealed elevated CPITN mean values for OLP patients compared to the healthy control group [32].

Sjögren's syndrome is a chronic autoimmune disease which affects the secretory glands of the body, such as lachrymal and salivary glands. The impaired function of these glands results in dried eyes and mouth, with negative consequences on the patient's life quality. As the saliva flow into the mouth is decreased or even absent in more advanced stages of the disease, the entire oral cavity is affected by different pathological events as described by Fox [33]. The teeth are no longer protected against bacteria by salivary components, and the dental plaque is no longer removed by the saliva flow. This triggers the progress toward dental caries and a shift in the oral mucosa which becomes more fragile and vulnerable to trauma and infection. As the salivary glands try to compensate for the lack of saliva production, they grow in size and distort the patient's facial appearance. Even though the pathological pathways of Sjögren's syndrome are not fully depicted, the possible autoimmune nature of the condition can be closely linked to viral infection. Despite the negative impact of the reduced salivary flow on the oral cavity, a direct connection between Sjögren's syndrome and periodontal disease has not yet been proven. A study by Kuru et al. found no significant differences between the periodontal status of such patients and healthy individuals, in terms of clinical and bacterial elements such as plaque sample microbiological analysis and characteristics of the periodontal lesions [34]. Another study by Boutsi et al. pointed that Sjögren's syndrome's patients with no other comorbidities expressed a periodontal status that was not significantly different and displayed even better oral hygiene than control subjects [35].

Oral-squamous-cell carcinoma, the most frequent type of oral cancer, is another HCV infection extrahepatic manifestation. Along with behavioral elements such as tobacco and alcohol intake, viral infection is considered an important risk factor for the development of oral cancer that is often derived from premalignant or potential malignant lesions of the oral mucosa such as leukoplasia, erythroplasia, and oral lichen planus. Nevertheless, for HCV-infected patients, the development of oral cancer has been reported even in the absence of such premalignant lesions [36]. A systematic review by Javed and Warnakulasuriya suggests that periodontal disease can boost the risk for oral cancer [37]. But even with a narrow increase, the importance is even greater if other mutual risk factors for both conditions such as tobacco and alcohol consumption are taken into consideration. In addition, the results of a study by Narayan et al. showed that oral-squamous-cell carcinoma and periodontal status are directly proportional in both clinical and microbiological aspects [38]. However, further research is required studying the periodontal status in HCV-infected patients with oral EHMs as the complexity of the subject needs to be closely analyzed by various medical specialties.

## 3. HCV Infection Transmission: Periodontal Disease and Oral Fluids

### 3.1. Saliva

HCV infection occurs by coming into contact with RNA viral molecules of infected patients. These molecules are carried by the patient's blood and can be found either inside blood products (like blood transfusions from untested donors) or on various objects after infected blood contact (like syringe needles) which are used by different individuals all together (like in the case of drug-addicted groups). The spread of the virus is even more difficult to combat as it can also be transmitted through unprotected sexual contacts, when one partner is infected but unaware of this fact because the disease is not clinically active in its first stages. The virus can also be transmitted through poorly sterilized needles used in tattooing and piercing [8].

The virus has a strong lymphotropism and can be found inside mononuclear blood cells. Therefore, it can be traced in fluids that contain such cells, like saliva [39–43]. In addition, despite the main parenteral way of transmission, HCV infection has also been reported in patients who were not exposed to needles, blood transfusions, or infected partners, which may suggest the existence of possible alternative transmission vectors [44]. As such, some studies have tested the possibility of infectious viral RNA molecules to be present in other body fluids than the blood [16, 45], the prevalence of such molecules in saliva being found inconsistent [46, 47].

Caldeira et al. conducted a study in which they recorded 36.8% positive HCV RNA saliva samples in the absence of anti-HCV serological antibodies and 23.5% positive HCV RNA saliva samples along with existent anti-HCV serological antibodies, all samples originating from infected patients [48]. Nevertheless, due to the fact that the HCV requires a host cell to replicate inside (targeting both hepatocytes and peripheral blood cells), the rates of viral RNA identification in saliva varied to a significant degree, as the variability of viral molecules' presence in peripheral blood cells found in saliva can have an important impact on the detection results [49–53]. The replication process of viral RNA requires the creation of negative-stranded intermediate RNA. While some studies have found no trances of such negative-stranded RNA in saliva samples [54], this type of RNA was found in the salivary gland tissue samples of infected patients also suffering from sialadenitis [55].

The biological mechanisms which enable the virus to exist in saliva are so far unclear. Even though the virus is mainly considered to be hepatotropic, there are scientific results suggesting that its replication could also take place inside peripheral mononuclear blood cells found in the submandibular salivary glands. This fact is supported by the involvement of the virus in the development of conditions such as sialadenitis and Sjögren's syndrome [56]. Arrieta et al. conducted an in situ study on salivary glands' epithelial cells originating from patients infected with HCV and suffering from sialadenitis or Sjögren syndrome. The results of the research reported a prevalence of HCV-infected cells in saliva ranging between 25% and 48.8% [55].

The probability of saliva being a vector for HCV infections has also been discussed in several case studies, addressing both, clinical reports of patients that had previously been bitten by infected HCV persons, and experimental research [57, 58]. However, studies using the polymerase chain reaction (PCR) method and trying to detect copies of viral RNA in saliva samples have not proven the infectious potential of these viral copies, the prevalence of viral RNA being extremely different [59, 60].

The inconsistent results of studies targeting the detection of viral RNA in HCV-infected patients' saliva can be explained through the origin of the virus as being the gingival crevicular fluid, more exactly the mononuclear blood cells found in this fluid. Therefore, periodontal status might be considered a contributing factor for the presence of HCV in saliva [61]. While the aspartate transaminase (AST) salivary levels have recorded significantly higher levels in samples from patients with chronic periodontal disease and the values of AST correlate to the degree of periodontal tissue injury [62], viral RNA molecules have been found in saliva samples of HCV patients regardless of their periodontal status [63].

### 3.2. Gingival Crevicular Fluid

The potential of HCV antigens and antibodies to be present in GCF has also been studied. The GCF is a particular oral liquid, as despite being derived from the internal environment (from serum) it is secreted in an outside space of the body (the gingival sulcus) and further away into the open oral cavity. Thus, its particularities in composition (inflammatory markers) and behavior (flow increase during inflammation) make the GCF a study-worthy subject on the matter of bidirectional links between periodontal disease and HCV infection. The fluid contains bacterial plaque components, inflammatory cells of the immune system, traces of connective tissue, and other serum factors [64].

Viral RNA and anti-HCV antibodies have been detected in GCF samples collected from HCV-infected patients [65–67]. The viral molecules are able to pass down into saliva, thus making the GCF the source of contamination of HCV-infected patients' saliva.

Periodontal inflammation, under the form of either gingivitis or periodontitis, causes a rise in gingival crevicular fluid flow and more frequent gingival bleeding. As a result, the virus can migrate more easily from the bloodstream into the gingival crevicular fluid and further away into the saliva, as it is being carried by peripheral mononuclear blood cells [68]. One study found a prevalence of 59% for HCV RNA in the GCF of HCV-infected patients [67]. Another study's results pointed an 83.72% prevalence rate for anti-HCV antibodies in the gingival fluid of infected patients, after using a quick-detection test [69]. Suzuki et al. showed that higher concentrations of HCV RNA were found in the GCF rather than in saliva for 77% of the infected patients enrolled in the study, while in 78% of the cases, the viral genetic material was found in the GCF even if it was absent in the saliva of infected patients [68]. The theory that the source for HCV RNA and antibodies in the oral cavity is in fact the gingival crevicular fluid is also sustained by Montebugnoli and Dolci's research [65]. Nevertheless, the efficiency of viral transmission via oral fluids could be dependent on the viral load, meaning that quantitative studies are further needed in order to assess the potential risk of nonparenteral ways of infection with HCV.

As far as the current scientific data goes, it appears that the best answer to the presence of RNA HCV molecules in the gingival fluid refers to infected leukocytes [70] that contain viral strains and get transferred from the bloodstream into the gingival fluid, mainly in the case of gingival inflammation. Thus, the subject still requires further research that should focus on the component particularities, both cellular and molecular, of HCV-infected patients' gingival fluid.

### 3.3. Implications of Viral RNA Presence in Oral Fluids

The detection of HCV RNA molecules in human body fluids other than the blood is subject to influence by sampling and analysis methods. Testing saliva samples for antigens or antibodies has revealed a similar or even higher specificity in comparison to testing serum, as suggested by studies assessing the efficiency of standard diagnosis tools for HCV infection and more recent ones such as HCV RNA oral detection kits for saliva, while also offering ways in which this less invasive method could be improved [66, 71]. The most frequent investigation methods include the use of the ELISA (enzyme-linked immunosorbent assay) test for the detection of anti-HCV antibodies in serum [72]. In contrast, saliva is easier to sample, an aspect which can facilitate and improve the screening of possible infected patients, thus having an impact on clinical, economic, and epidemiologic factors addressing the issue of HCV infection [71]. However, even if in terms of specificity the two fluids are comparable, in terms of sensibility, the tests used on blood serum are more appropriate as there is a lower concentration of antibodies in saliva. This downturn can be improved by adjustment of the protocol such as sample expansions, dilution reductions, and incubation time augmentation [66]. De Cock et al. showed that a modified ELISA method can be used as a tool for running epidemiologic studies for anti-HCV antibody detection within oral fluids, as it exhibits a satisfactory degree of specificity [73].

There are studies [74, 75] claiming an 80–87% sensitivity and a 100% specificity for the detection of anti-HCV antibodies in oral fluids in comparison to100% for blood serum. Although there is no significant correlation between the viral load and the levels of the HCV in the saliva, patients with low levels of serum HCV pointed out a minor viral RNA concentration in the saliva. Nevertheless, Hermida et al. identified a significant correlation between the serum viremia and the concentration of viral RNA in saliva [76]. This may have an important impact among caregivers, suggesting the need of further epidemiologic studies [65]. However, the data is still controversial, as some authors revealed that the presence of viral RNA in the saliva is not dependent on serum viremia or the presence of other oral conditions [77].

The involvement of HCV by itself in salivary dysfunction is still questionable [78, 79], but the association of hepatitis C infection with Sjögren's syndrome is recognized as a cause for salivary hypofunction, the reduced rate of salivary flow potentially being the cause of most dental and oral conditions in patients infected with HCV, as well as the insulin resistance which these patients manifest, thus being prone to the development of periodontal disease. The management of HCV-infected patients is a real challenge due to the pathological particularities that these patients imply and to the impact that the infection has on treatment planning and prognostic.

## 4. Periodontal Disease: Impact on HCV Infection

The liver function is mainly evaluated by means of serological testing of two enzymes—aspartate aminotransferase (AST) and alanine aminotransferase (ALT), their levels being elevated in hepatic distress and inflammation. The AST salivary levels have recorded significantly higher levels in samples from patients with chronic periodontal disease as shown in a study by Kudva et al., and what is more, the values of salivary AST correlate to the degree of periodontal tissue injury [62].

The GCF represents either a serous transudate or an inflammatory exudate, depending on the status of the periodontal tissues and can easily be collected from the gingival sulcus surrounding each tooth. Various components of the GCF, such as cytokines and metalloproteinases (MMPs), like IL-1, IL-6, IL-12, TNF-alpha, VEGF, and MMP9 can be considered periodontal inflammatory markers, their identification in the GCF being used to study the common pathways between periodontal disease and other conditions such as type 1 and 2 diabetes, heart disease, and rheumatoid arthritis [80–82], since the presence of several of these biomarkers has been detected in serum and gingival epithelium of the periodontal-affected patients with some comorbidities [82–85].

The scientific data justifies the need for studies assessing the different proinflammatory components of GCF in patients with HCV infection or with HCV infection and periodontal disease. However, there are studies finding a high level of some of these markers like interleukins IL-1, IL-6 and interferon IFN-gamma in the serum of HCV-infected patients and also in the serum and GCF of patients with periodontal infection [86–88]. Further studies are required to assess the link between other inflammatory markers found in the gingival fluid and the HCV infection [20] and thus to investigate if the HCV-infected patients would have a higher risk to develop periodontal injury or if periodontal-compromised patients with type C hepatitis could have a higher expression of other liver-specific biomarkers, along with some insights into the underlying mechanism.

It was shown that the levels of AST in the GCF of patients with periodontal disease are elevated and correlated with the periodontal disease activity [89, 90], recording a declining trend after periodontal treatment and associating clinical parameter improvement [91]. Moreover, in nonalcoholic fatty liver disease (NAFLD) subjects, there is an important liver biomarker improvement (expressed as ALT and AST activity) following efficient periodontal therapy. Thus, the periodontal inflammation determined by *P*. *gingivalis* in NAFLD patients could be a risk factor for the exacerbation of NAFLD, and the periodontal treatment could be useful in the management of NAFLD subjects [92]. The mechanisms that have been considered to be responsible for the implication of periodontitis in different liver diseases were related to bacterial agents, proinflammatory mediators, and oxidative stress. Together with cytokines and chemokines, other molecules such as heat shock proteins (HSPs) could be produced as a result of periodontal bacterial aggression and could consequently trigger hepatic inflammation [17]. Together with the pathological mechanism proposed in Section 2.1 of this review article (IR involvement), the study of TNF-alpha and other proinflammatory cytokines could provide a thorough explanation for the possible chronic hepatitis C infection and periodontal disease connection. Comprehensive understanding of the bidirectional pathological mechanism that joins the two conditions is required in both periodontology and hepatology fields in order to provide proper diagnostic and treatment protocols.

## 5. Conclusion

Through different means, HCV infection causes a decline in the oral health of infected patients. Whether considered by some authors to be connected to extrahepatic manifestations of the infection or derived from the systemic implications (malfunction of the immune system) or caused by the patients' behavior (poor oral hygiene and diet), the periodontal impairment of HCV-infected patients accounts for an important cutback of life quality and welfare. Moreover, viral RNA molecules and antibodies have been identified in patients' saliva and gingival fluid, raising further questions about the disease's transmission and detection.

The study of the common immunological link between type C viral hepatitis and periodontal health can be the subject of further research, not only in what concerns the clinical parameters but also through identification of specific markers in GCF, exploring the merger of the pathological mechanisms, possibly related to IR and chronic inflammatory process. This may lead to a better understanding upon whether this relationship is bidirectional and implicitly upon the underlying mechanisms that induce progression toward their pathologies. This bidirectional relationship between periodontal disease and HCV infection has far-reaching implications for both patients and medical practitioners, either periodontologists or hepatologists.
